# C1QA and COMP: plasma-based biomarkers for early diagnosis of pancreatic neuroendocrine tumors

**DOI:** 10.1038/s41598-023-48323-x

**Published:** 2023-11-29

**Authors:** Priya Kumari Gorai, Prahalad Singh Bharti, Shashi Kumar, Girish H. Rajacharya, Sabyasachi Bandyopadhyay, Sujoy Pal, Renu Dhingra, Rakesh Kumar, Fredrik Nikolajeff, Saroj Kumar, Neerja Rani

**Affiliations:** 1 Department of Anatomy, All India Institute of Medical Sciences, New Delhi, India; 2 Department of Biophysics, All India Institute of Medical Sciences, New Delhi, India; 3 Department of Metabolic Engineering, International Centre for Genetic Engineering and Biotechnology, New Delhi, India; 4 Centralized Core Research Facility, All India Institute of Medical Sciences, New Delhi, India; 5 Department of GI Surgery, All India Institute of Medical Sciences, New Delhi, India; 6 Department of Nuclear Medicine, All India Institute of Medical Sciences, New Delhi, India; 7 Department of Health Science, Lulea University of Technology, Luleå, Sweden

**Keywords:** Tumour biomarkers, Pancreatic cancer, Proteomics

## Abstract

Pancreatic Neuroendocrine tumors (PanNET) are challenging to diagnose and often detected at advanced stages due to a lack of specific and sensitive biomarkers. This study utilized proteomics as a valuable approach for cancer biomarker discovery; therefore, mass spectrometry-based proteomic profiling was conducted on plasma samples from 12 subjects (3 controls; 5 Grade I, 4 Grade II PanNET patients) to identify potential proteins capable of effectively distinguishing PanNET from healthy controls. Data are available via ProteomeXchange with the identifier PXD045045. 13.2% of proteins were uniquely identified in PanNET, while 60% were commonly expressed in PanNET and controls. 17 proteins exhibiting significant differential expression between PanNET and controls were identified with downstream analysis. Further, 5 proteins (C1QA, COMP, HSP90B1, ITGA2B, and FN1) were selected by pathway analysis and were validated using Western blot analysis. Significant downregulation of C1QA (p = 0.001: within groups, 0.03: control vs. grade I, 0.0013: grade I vs. grade II) and COMP (p = 0.011: within groups, 0.019: control vs grade I) were observed in PanNET Grade I & II than in controls. Subsequently, ELISA on 38 samples revealed significant downregulation of C1QA and COMP with increasing disease severity. This study shows the potential of C1QA and COMP in the early detection of PanNET, highlighting their role in the search for early-stage (Grade-I and Grade-II) diagnostic markers and therapeutic targets for PanNET.

## Introduction

Neuroendocrine tumors (NETs) encompass a collection of tumors that arise from neuroendocrine cells and can be detected across various organs, with notable prevalence in the lung, digestive tract, and pancreas^1^. NETs rarely occur in 2 cases per 100,000 individuals, representing approximately 0.5% of all tumors^2,3^. The clinical features of neuroendocrine tumors (NETs) in the Indian population exhibit significant variations compared to Western nations, particularly regarding the distribution of neuroendocrine tumors by anatomical site and tumor type. Recent studies conducted in India have revealed that the pancreas (approx. 35%) stands as the primary and prevailing site of origin for neuroendocrine tumors (NETs)^3,4^.

The term “pancreatic neuroendocrine tumors” (PanNET) refers to a broad category of neoplasms that develop from neuroendocrine cells in the pancreas. These tumors stand out from other pancreatic cancers due to their distinctive clinical, histomorphologic, and prognostic characteristics^5–7^. PanNET can differ significantly in their clinical characteristics. They might be benign, slowly expanding tumors with no symptoms, or they can be more aggressive varieties that result in hormonal imbalances and different clinical disorders^8^.

Various biochemical tests, such as complete blood count (CBC), serum calcium, renal and liver function tests (RFT/LFT), chromogranin A, neuron-specific enolase, pancreatic polypeptide, pancreastatin, CA 19-9, serotonin derivatives (5-hydroxyindoleacetic acid), insulin, glucagon, gastrin-1, and vasoactive intestinal peptide, play a crucial role in screening, diagnosis and prognosis of PanNETs patients ^9,10^. The available laboratory tests lack sensitivity and specificity in diagnosing pancreatic neuroendocrine tumors. Additionally, the imaging techniques employed for diagnosis, such as endoscopic ultrasound, CT scans, X-rays, Octreotide scintigraphy, [68Ga] Ga-DOTATATE PET (Dota-Octreotate Positron Emission Tomography), and [18F]-FDG (Fluorodeoxyglucose)-PET scans, are invasive and expensive. Moreover, these advanced imaging techniques may not be accessible in all medical centers, making them particularly inaccessible for individuals in developing countries^11–14^. Therefore, there is an urgent need for more specific, sensitive, and cheap biomarkers for early screening and diagnosis of this disease.

Proteomics carries significant potential for advancements in molecular medicine, as evidenced by studies exploring its novel perspectives in cancer research. A notable aspect of proteomics is its promise in discovering biomarkers and tumor markers, which can be helpful in the early detection and diagnosis of various diseases, with a particular focus on cancer^15–17^. Additionally, discovering specific protein markers can aid in creating personalized medicines that maximize therapeutic effectiveness while minimizing adverse effects for each patient^16,18^. So, proteomics continues to be the preferred method for conducting biochemical investigations on several cancers, yielding crucial insights such as protein profiles, protein levels, modification sites, and protein interactions ^18,19^.

Among the applications of proteomic techniques, Mass spectrometry offers significant advancements in proteomic studies, particularly in enhancing signal specificity by effectively eliminating false-positive results during database searching. It enables the quantification and identification of proteins within complex protein mixtures, analysis of protein–protein interactions, investigation of post-translational modifications, Structural proteomics, and the identification of differential protein modifications^20^. Proteomics has been invaluable in discovering numerous cancer biomarkers such as breast, esophageal, Gastric, lung, colorectal, liver, etc^21^. Proteomics analysis has been reported in cases with neuroendocrine tumors, greatly aiding the understanding of neuroendocrine tumors (NETs) pathogenesis.

The current investigation examined the proteomic profiles of plasma samples obtained from individuals with PanNET (stage I and stage II) and healthy individuals serving as controls. This is the first study focuses on the plasma sample of PanNET and healthy individuals. Our objective was to identify particular plasma proteins that could prove beneficial in detecting PanNET.

## Results

### Demographical and clinical characteristics of pancreatic neuroendocrine tumor (PanNET) patients and healthy control subjects

**Figure 1:**
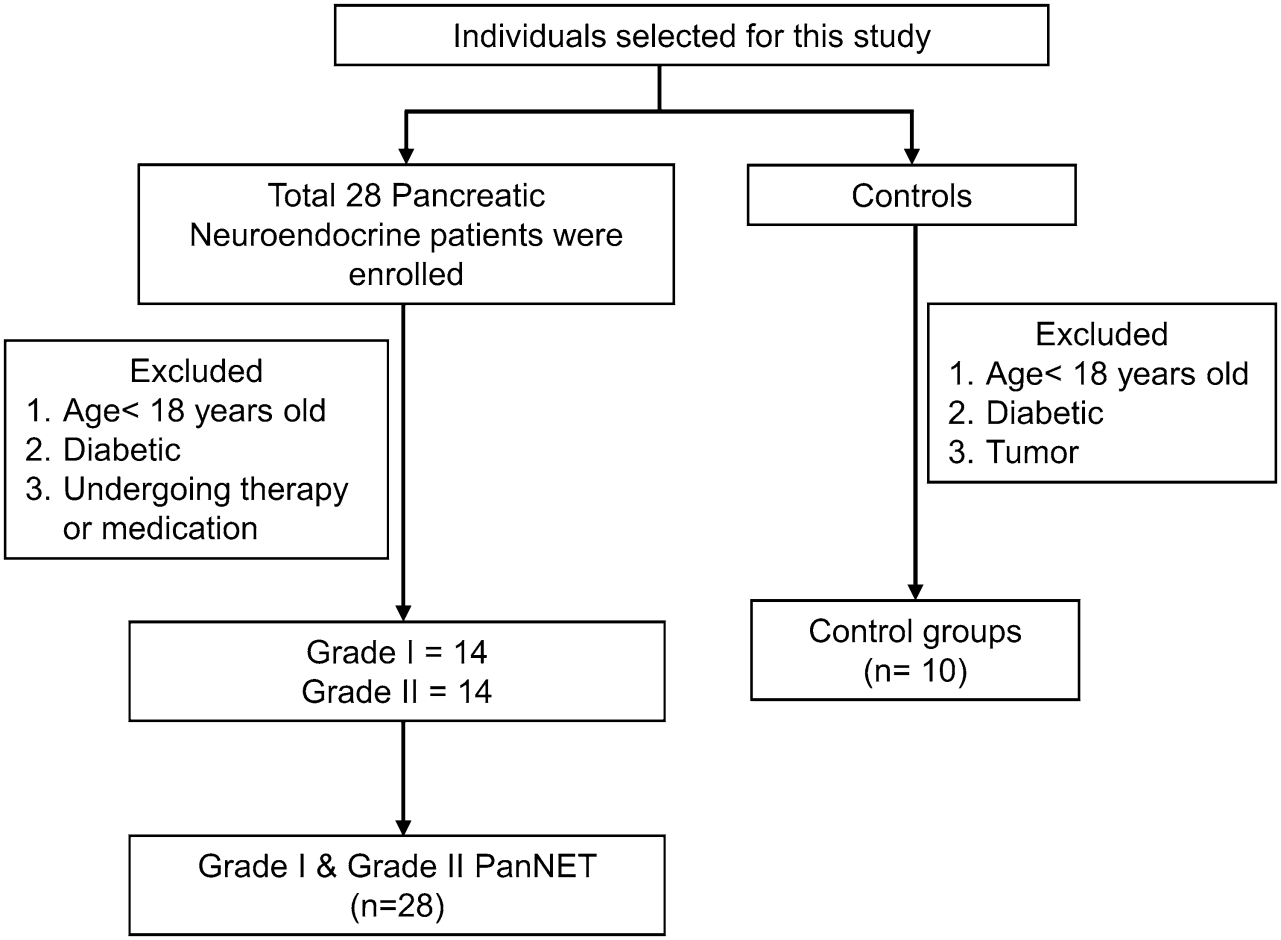
A flowchart illustrating the process of selecting PanNET patients.

The mean age of PanNET patients is 41.89 ± 2.75 years (age range 20–70 years). In this study, out of 28 PanNET patients, 15 were male, and 13 were female, whereas in 10 healthy controls, 6 subjects were male and 4 were female. WHO grading of PanNET patients was done according to the 2017 WHO classification. Out of 28 patients, approx. 50% are with grade-I tumors, and 50% are with grade-II tumors; however, > 50% are found to be diagnosed at the metastatic stage (Fig. 1). 50% of patients have Ki67 index > 3%. The detailed history of the patients has been mentioned in Table 1**.**

**Table 1:** Demographic details of PanNET patients.

Characteristics		No. of patients (n = 28)	Percentage %
Age in years	Mean ± SEM	41.89 ± 2.75 (20–70)	
	20–50	21	75%
	Above 50	07	25%
Sex	Male	15	53.5%
	Female	13	46.4%
Metastasis		13	46.4%
Stage/Grade	Grade I PanNET	14	50%
	Grade II PanNET	14	50%
	Grade III PanNET	-	–
Ki67 index	1–2%	14	50%
	3–5%	11	39.28%
	> 5%	03	10.71%

### Proteomic profiles of PanNET plasma

**Figure 2:**
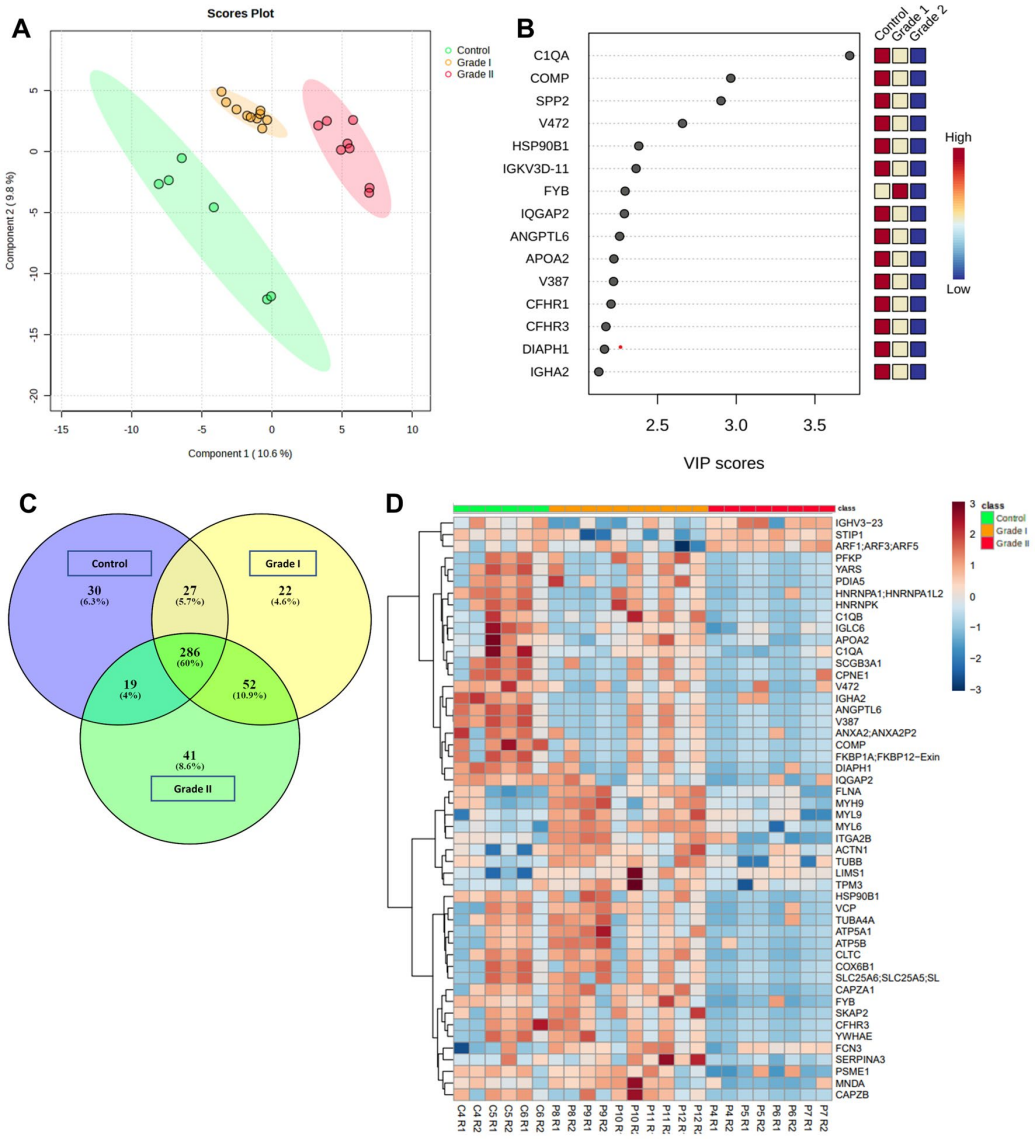
Analysis of proteomics data. (**A**) The figure illustrates the scores plots of components one and two in a Partial Least Squares discriminant analysis (PLS-DA), explicitly comparing the clustering of PanNET (Grade I and II) and control samples. (**B**) The plot of the top 15 variable importance in projection (VIP) scores (> 1.5) was obtained from the analysis of PLS-DA. (**C**) Venn diagram created using the program Venny (https://bioinfogp.cnb.csic.es/tools/venny), illustrating the total count of proteins that exhibited differential expression in each Group (Grade I, Grade II, and Control). (**D**) The heat map displays the differential proteins, where protein expression values have been log2-normalized. Cluster analysis was conducted using Z-score protein intensities for proteins with a significance level of p < 0.05. In the heat map, red indicates a high expression level, while blue indicates a low expression level.

Partial least squares-discriminant analysis (PLS-DA) modeling used proteomics data to distinguish the distinct separation between Patients (Grade I and Grade II PanNET) and controls. This analysis simultaneously identified proteins whose expression contributed to the discrimination among the three groups. Figure 2A and B show PLSDA and the associated VIP classification model. Significant separation of Grade II PanNET from Grade I and Control in Component 1 was observed via the PLSDA score plot. Each symbol indicates data from each group. Ellipses signify a 95% confidence interval. The 15 proteins with the highest VIP scores are displayed in VIP score plots. Figure 2C illustrates the total count of proteins that exhibited differential expression in each Group (Grade I, Grade II, and Control). The web-based tool Venny 2.1 software was utilized to generate and interpret the Venn diagram based on the proteomics data, which shows that 60% of the proteins were commonly expressed in patients as well as controls, whereas 13.2% of proteins were uniquely reported in PanNET patients (Grade I and Grade II). Figure 2D presents heatmaps illustrating the differences between detected metabolites in three groups: PanNET Grade I, PanNET Grade II, and the control. The heatmap shows that the Control group exhibits minimal differences in metabolic profiles compared to the Grade I PanNET group. Subtle differences between the PanNET Grade I and Grade II groups can also be observed. However, notable changes across a range of proteins are evident when comparing the metabolic profiles of the Control group to the Grade II PanNET group. (GI Vs Control; GII Vs Control then GI Vs GII). Furthermore, in the comparison between PanNET Grade I and Grade II, a significant decrease in a cluster of metabolites can be observed in cases of healthy control groups.

### Identification of differentially expressed proteins in plasma of PanNET and control

**Figure 3:**
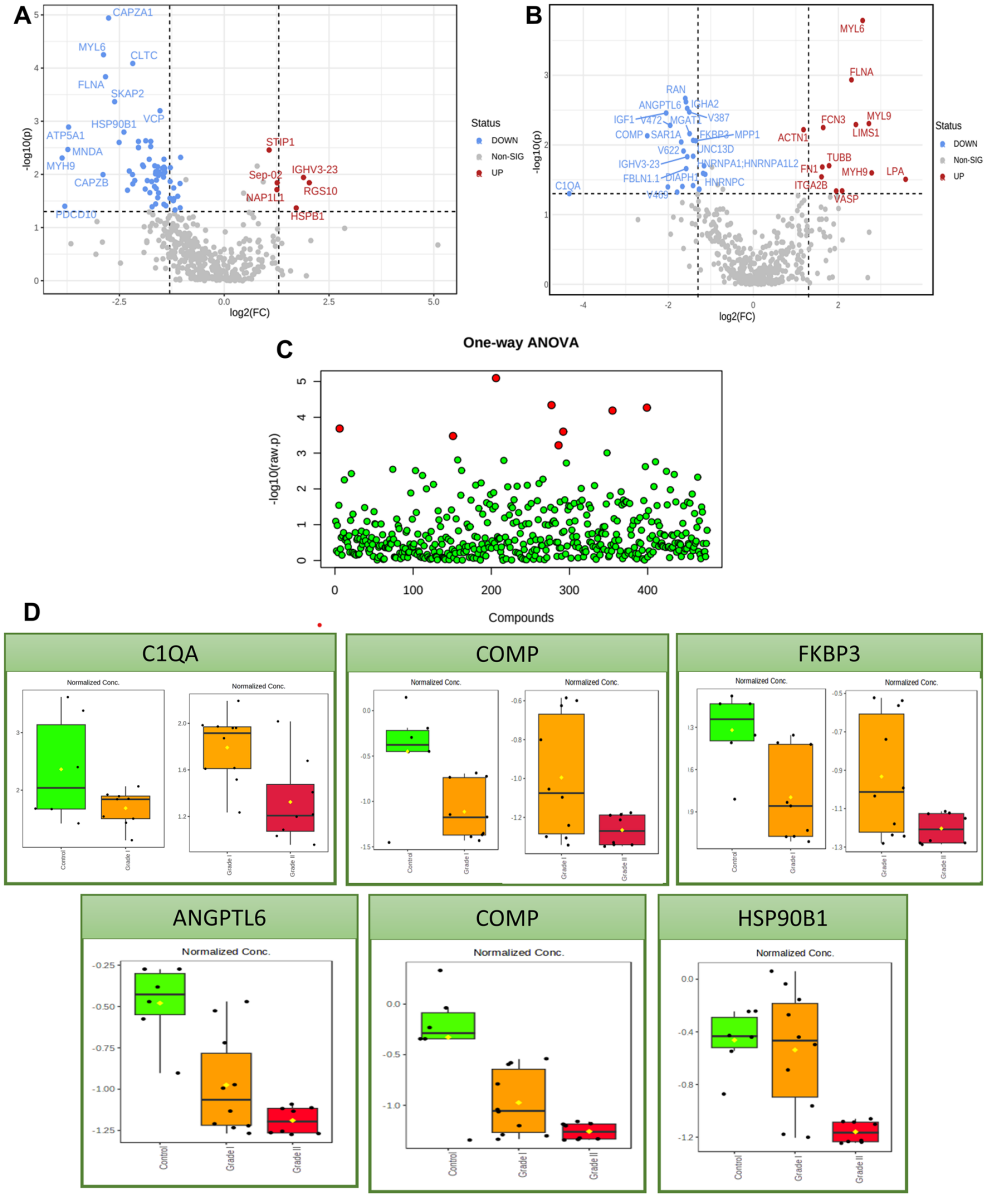
Quantitative proteomics of Plasma proteins. The volcano plot shows the Log2 fold changes and corresponding p-values of all proteins between PanNET Grade I and control (**A**) and between Grade II and Grade I PanNET (**B**). In the plot, proteins exhibiting upregulation with more than a twofold change and a p-value < 0.05 are depicted as red circles. In contrast, green circles represent proteins displaying downregulation with the same fold change and p-value. Additionally, grey circles indicate plasma proteins that did not demonstrate statistically significant differences. (**C**) The plot depicting the Analysis of Variance (ANOVA) displays the significantly (p < 0.05) detected metabolites when comparing the three groups (Grade I and Grade II PanNET) with the control group. (**D**) The box plots illustrate the distribution of relative protein abundance (commonly downregulated in Volcano Plot and ANOVA) in the plasma of subjects with PanNET (Grade I and II) compared to control subjects.

Univariate and multivariate statistical analyses were further performed to assess the patients' and controls' metabolic profile differences. Figure 3A illustrates the proteomics data comparing Grade I PanNET to the control group, while Fig. 3B represents the comparison between Grade II and Grade I PanNET. Both figures depict volcano plots, where each point corresponds to a unique protein showing a significant two-fold change (x-axis) and robust statistical significance (p ≤ 0.05). This study identified 27 proteins that showed downregulation and 13 that exhibited upregulation in Grade I PanNET compared to the control group. On the other hand, Grade II PanNET demonstrated 69 downregulated proteins and 6 upregulated proteins compared to Grade I PanNET (Supplementary File, Table S1). We identified 11 common proteins in both groups, indicating their presence in both Grade I PanNET compared to the control group and Grade II PanNET compared to Grade I PanNET. Additionally, we found 8 proteins that exhibited downregulation in both groups, specifically in Grade I PanNET concerning the control group and Grade II PanNET concerning Grade I PanNET.

The analysis of variance (ANOVA) identified 8 metabolites that exhibited significant differences (p < 0.05) between Grade I and II PanNET and the control group (Fig. 3C). Figure 3D displays representative proteins selected from the ANOVA and Volcano plot analysis. Among the 11 proteins identified in the volcano plot and the 8 proteins detected in the ANOVA, six proteins highlighted in Fig. 3D exhibit significant downregulation as the severity of the disease increases.

### Pathway analysis with STRING databases

**Figure 4:**
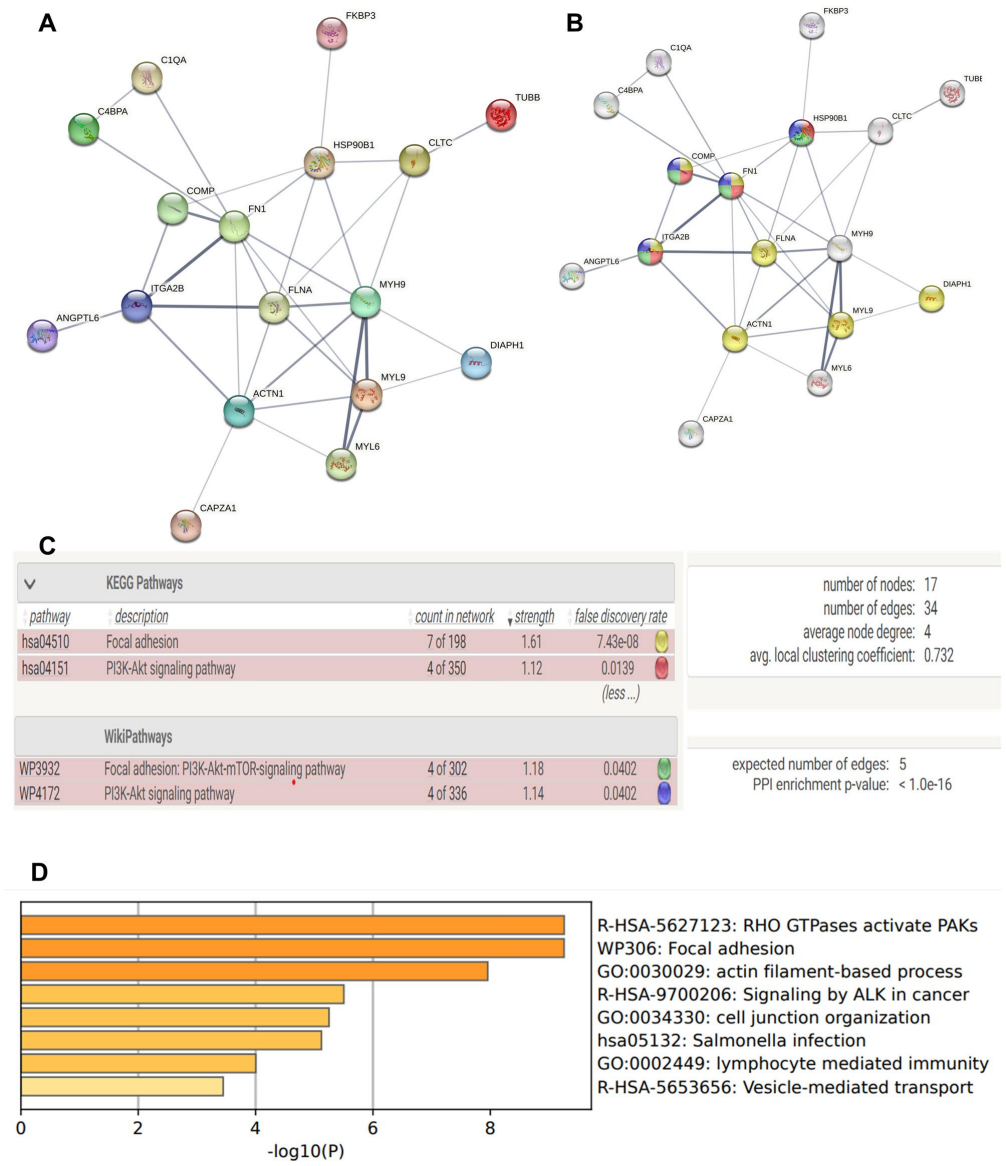
Protein–protein interaction for the differentially expressed proteins identified through LC–MS-based proteomics analysis. The interactions were analyzed using the STRING software. (**A**) In the network, proteins are represented as nodes, and the thickness of the connecting lines indicates the degree of association between the proteins. (**B**, **C**) The yellow and green nodes denote proteins implicated in the Focal adhesion pathway, whereas red and blue nodes denote proteins implicated in the PI3K-Akt Signalling pathway. (**D**) Top dysregulated pathway by Gene Ontology (GO) analysis.

The differentially expressed proteins discovered by LC–MS-based proteomics were searched for in the STRING protein–protein interaction database to look for known and anticipated interactions. To identify pathways distinguishing PanNET from healthy controls, we categorized the proteins based on Gene Ontology (GO) terms using DAVID software. Based on our findings, we constructed a protein–protein interaction network (Fig. 4A and B). These proteins are shown to be highly confidently involved (p values < 1.0 E−16) networks consisting of 17 nodes and 34 edges. Pathway analysis through String PPI database showed that these proteins were associated with actin cross-links, platelet aggregation and degranulation, angiogenesis, non-metabolic focal adhesion, and PI3K-Akt signaling pathways (Fig. 4C and D). 4 proteins (COMP, HSP90, ITGA2B, and FN1) out of all 17 proteins were associated with top two focal adhesion, and PI3K-Akt signaling pathways when assessed by pathway analysis programs (Fig. 4B and C). The top eight GO pathways for the differentially regulated proteins are shown in Fig. 4D. The pathways analysis revealed that the proteins exhibiting differential regulation were primarily linked to focal adhesion and PI3K-Akt signaling pathways, both recognized as crucial pathways in tumor formation.

### Selection and validation of selected differentially expressed proteins for PanNET

The biomarker selection process was carried out in coarse selection and fine selection. A volcano plot, ANOVA, and VIP (Variable Importance in Projection) values were generated through PLS-DA (Partial Least Squares-Discriminant Analysis) modeling in the coarse selection step. In the fine selection step, significant proteins were identified by identifying the overlap between the results obtained from these two techniques and which are linked to cancer pathways.

**Figure 5:**
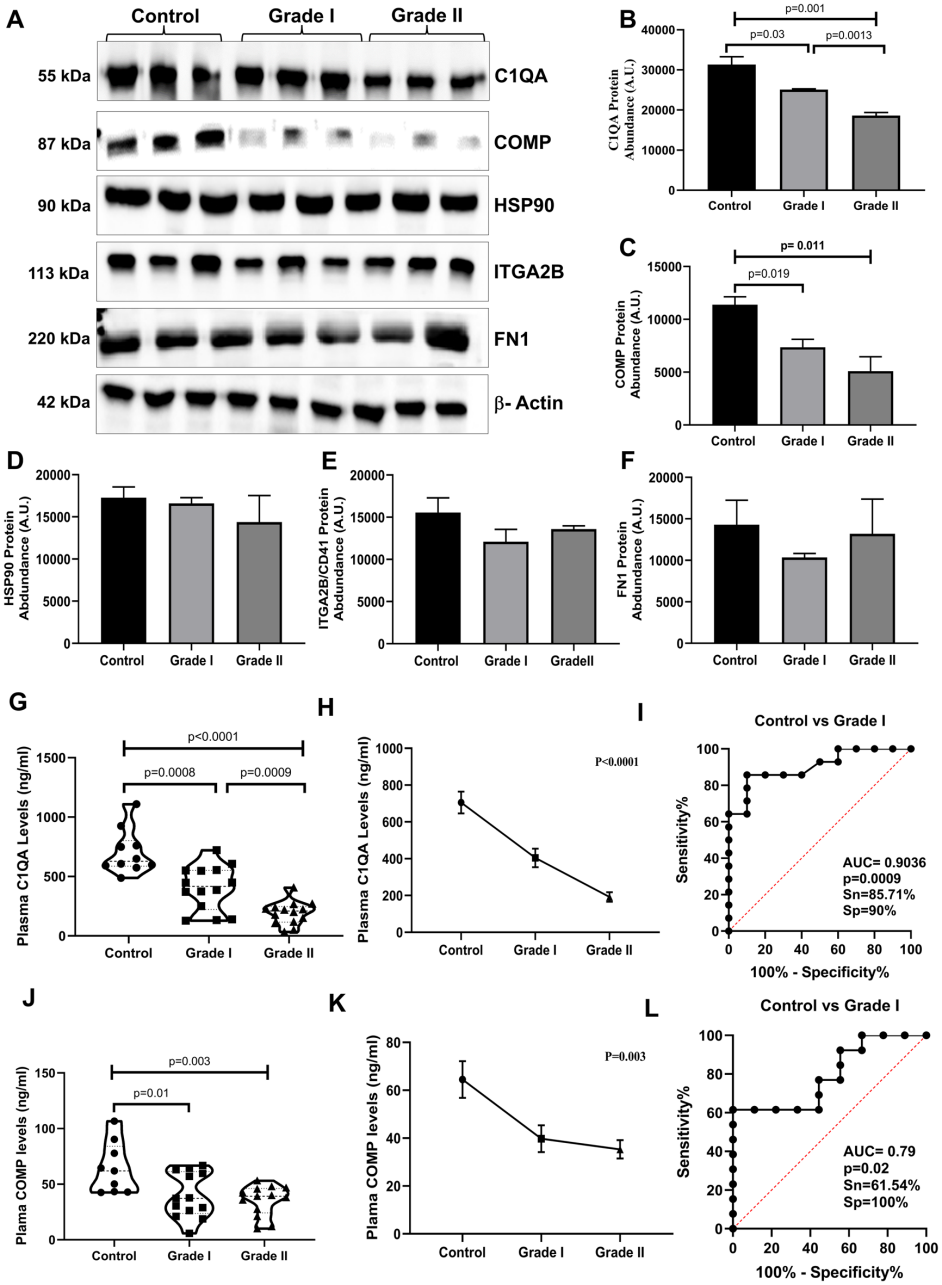
Validation of potential protein targets. Western blot profiling and densitometric analysis of final selected proteins (**A**). Densitometric analysis of (**B**) C1QA (p = 0.001, 0.03, 0.0013); (**C**) COMP protein (p = 0.011, 0.019), (**D**) HSP90B1, (**E**) ITGA2B/CD41, (**F**) FN1, (**G**, **H**) Sandwich ELISA for C1QA binding analysis (**I**) ROC curve analysis for plasma C1QA levels in Grade I PanNET vs controls (**J**, **K**) Sandwich ELISA for COMP analysis **(I)** ROC curve analysis for plasma COMP levels in Grade I PanNET vs controls.

For western blot analysis, five proteins (C1QA, COMP, HSP90B1, ITGA2B, and FN1) were selected (Fig. 5A). The western blot analyses demonstrated a notable downregulation of C1QA (Fig. 5B; Supplementary File, Fig. S2) and COMP (Fig. 5C; Supplementary File, Fig. S3) with the progression of disease severity, indicating lower expression levels in Grade I & II PanNET than controls. These findings were consistent with the results obtained from Proteomic analysis. When comparing the western blot analysis with the proteomics results for the other three proteins, HSP90B1, ITGA2B, and FN1 (Fig. 5D–F; Supplementary File, Figs. S4–S6), it was observed that the overall trends in expression changes were consistent between the two methods, but they did not reach statistical significance. β- actin was employed as an internal control for PanNETs and control (Fig. 5A; Supplementary File, Fig S7). Subsequently, C1QA and COMP were validated on a large sample size. An ELISA-based assay was employed to examine the concentration of C1QA and COMP in the plasma of both PanNET patients and control subjects (Fig. 5G, H, J and K). ROC curve analysis was also done for plasma C1QA and COMP level for PanNET patients (Grade I) and controls, obtaining AUC = 0.90 (p = 0.0009) with a sensitivity of 85.7% and specificity of 90% (Fig. 5I) for C1QA and AUC = 0.79 (p = 0.02) for COMP (Fig. 5L). As anticipated, the plasma of patients exhibited lower levels of C1QA and COMP compared to the healthy control group. Specifically, both PanNET grade I and grade II patients demonstrated reduced levels of C1QA in comparison to the control group, with concentrations of 404.2 ± 50.05 ng/mL, 192.2 ± 26.01 ng/mL, and 705.4 ± 59.53 ng/L, respectively. Similarly, for COMP, both PanNET grade I and grade II patients showed reduced levels compared to the control group, with concentrations of 39.79 ± 5.6 ng/mL, 35.33 ± 3.8 ng/mL, and 64.5 ± 7.6 ng/L, respectively.

## Discussion

Pancreatic Neuroendocrine tumors (PanNET) often show no symptoms or nonspecific ones, leading to late-stage diagnosis and poor prognosis. Early screening methods, are urgently needed for better treatment outcomes. Proteomics, a field of study focused on the comprehensive analysis of proteins, has emerged as a valuable tool in biomedical research^17,22^. Proteomics helps us understand diseases and find possible biomarkers by analysing the whole collection of proteins present in a biological sample. In the context of PanNET, plasma samples from various tumor stages (Grade I and Grade II) were analysed using proteomics, including controls without the disease. Plasma samples were subjected to mass spectrometry for quantification and identification proteins. The study compared Grade I PanNET, Grade II PanNET, and control groups to identify specific protein markers. Analysis revealed 17 candidates with significant expression differences between pancreatic NET individuals and controls. Dysregulated proteins were analysed using STRING and DAVID tools to identify interactions, enriched pathways, and associated diseases.

The pathways analysis indicated that these proteins with altered regulation were predominantly linked to focal adhesion and PI3K-Akt signaling pathways, recognized as pathways significant in cancer. Research has demonstrated that Focal Adhesion Kinase (FAK) plays a role in the survival of normal pancreatic islets through AKT activation. In the context of malignant transformation from islet cells to pancreatic neuroendocrine tumors (PanNETs), there is often overexpression of AKT and the presence of mutations in the AKT/mTOR pathway^23^. Additional investigations were conducted to validate potential cancer pathway-associated biomarkers and evaluate their clinical significance. C1QA and COMP, among the 5 candidates, proved to be confirmatory biomarkers with significant and consistent expression differences, holding promising potential for early pancreatic NET diagnosis.

Complement C1q serves as the primary activator of the classical pathway, but recent research indicates that C1q plays a role in the tumor microenvironment by promoting cancer development, even without complement activation. C1q has been detected in several cancers, including colon, lung, breast, pancreatic carcinoma, and melanoma. In melanoma cells, its presence has facilitated adhesion, migration, and proliferation^24^. Contrary to previous beliefs, some studies have proposed that C1q can trigger apoptosis and inhibit tumor growth, thus exerting tumor-suppressive effects. A recent study by Hong et al. observed that C1q, naturally expressed in the normal prostate, showed decreased levels in benign prostatic hyperplasia and prostate cancer cases. Similar to the above findings, in our study, C1QA exhibited a gradual downregulation with increased disease severity. This finding suggests that C1QA may be crucial in suppressing tumor development ^25–27^.

The extracellular matrix protein cartilage oligomeric matrix protein (COMP), a hallmark of cartilage metabolism, controls cellular phenotype during tissue creation and remodeling. Emerging research indicates that COMP significantly advances colon, breast, and prostate cancer^28,29^. It helps cell proliferation by triggering the Akt pathway, contributing to the progression of these cancers. COMP has been linked to tumor immune evasion in different types of cancer, including pancreatic adenocarcinoma^29,30^. This study observed an association between COMP and the Akt signaling pathway. However, contrary to expectations, the expression of COMP decreased as the severity of the disease increased. This intriguing finding suggests that COMP may have distinct roles in the context of pancreatic neuroendocrine tumors compared to other cancer types. The selection of C1QA and COMP as the preferred biomarkers implies that these proteins strongly associate with pancreatic NET and hold promise as indicators for early disease detection. Their differential expression in pancreatic NET patients compared to controls suggests that they may play a crucial role in the pathogenesis or progression of the disease.

**Figure 6:**
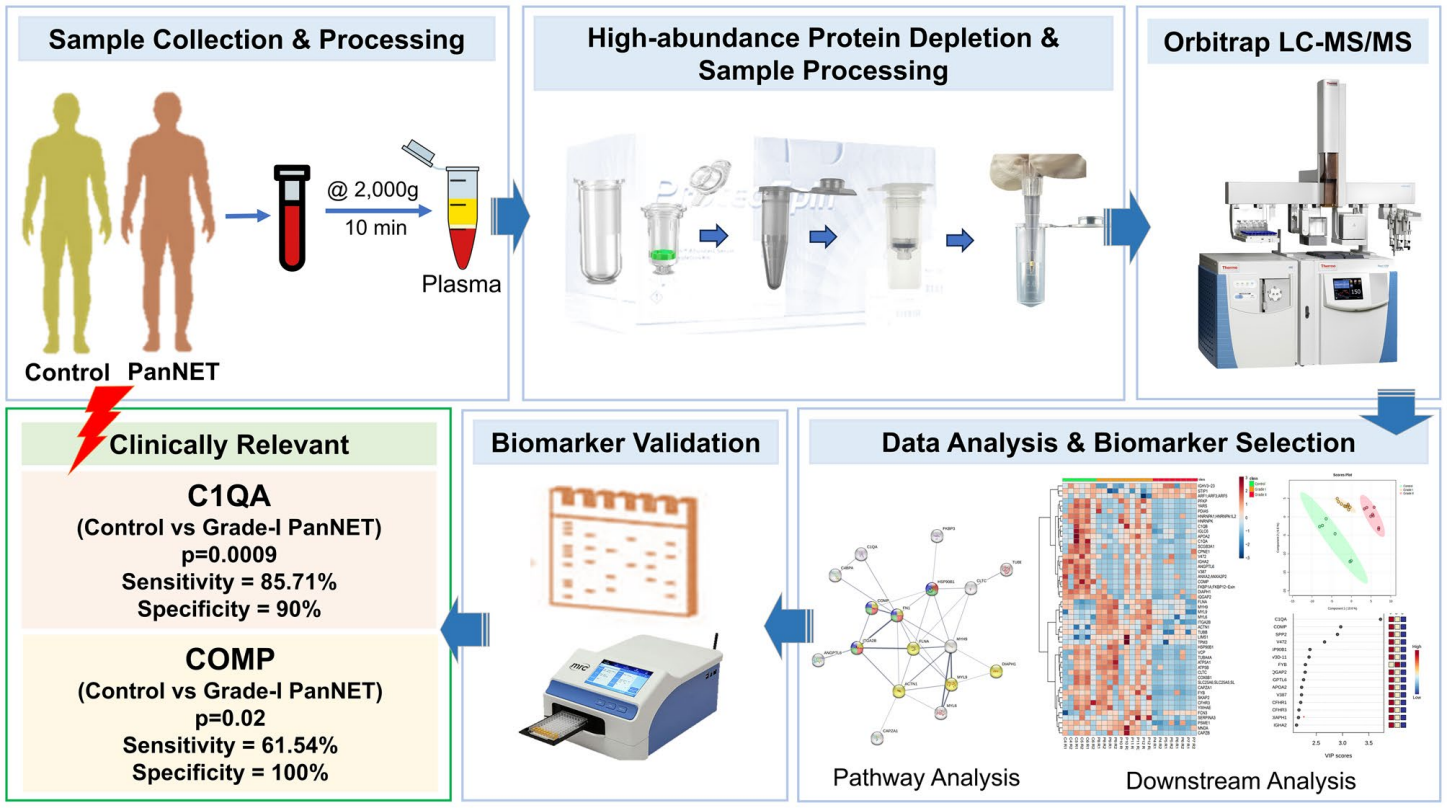
Figure illustrating a graphical summary of the paper.

In conclusion, this study shed light on the proteomic alterations associated with PanNET and identified novel biomarkers that could aid in early-stage detection, prognosis, or treatment monitoring by utilizing the power of proteomics. Our study demonstrated that plasma-derived proteins could assist as optimal minimal-invasive biomarkers for the early detection of PanNET (Fig. 6). This study provides valuable insights into the molecular mechanisms underlying PanNET by pinpointing these specific biomarkers and identifying C1QA and COMP as potential early-stage (Grade-I and Grade-II) biomarkers could open up new possibilities for developing targeted diagnostic approaches that could lead to earlier detection, improved prognosis and more effective treatment strategies for patients with PanNET. In summary, we presented a systematic approach for screening and identifying plasma-derived biomarkers for PanNET detection. Further validation studies and clinical trials will be necessary to confirm the utility of these biomarkers and will help develop new biomarkers for the disease.

## Material and methods

### Subjects

38 samples were gathered, including 28 from PanNET patients and 10 controls. The patients were recruited from the Gastrointestinal Surgery Department and Nuclear Medicine Department at AIIMS, New Delhi. The selection process did not impose sex-based restrictions and included individuals above 18 years. The selection criteria encompassed patients without treatment (such as surgery, chemotherapy, radiation, or peptide receptor therapy).

Ethical approval for the research was granted by the institutional ethics committee of All India Institute of Medical Sciences, New Delhi, India (Ethical Approval Number: IECPG-452/25.08.21). The methodologies used in this study aligned with the standards outlined in the Declaration of Helsinki. Given the rarity of the disease (with an incidence of 2 cases per 100,000 individuals), this preliminary investigation justifies using a small sample size to establish a proof-of-concept. Exclusions from the study involved patients with diabetes and those who declined to provide consent. To form a comparison, 10 normal healthy controls were selected, disregarding sex and ensuring they were non-diabetic and untreated. All the subjects were recruited for the study after obtaining the written informed consent form. A detailed written participant information sheet and participant informed consent form were provided to the subjects to participate in this study, and their signatures were obtained.

### Samples collection

5 mL of whole blood was withdrawn from the PanNET patients and healthy controls in the resting state. Subsequently, the blood samples underwent plasma separation through centrifugation at 2000 rpm for 10 min at 4 °C, and the resulting supernatant was collected. The clarified plasma samples were stored at − 80 °C until further experimentation.

### Sample preparation for LC–MS

A total of 12 samples (n = 3, Control; n = 5 Grade I and n = 4 Grade II PanNET) were used for Proteomic analysis. The BCA method was employed to determine the total protein content to prepare the sample, and a quantity of 100 µg of total protein was utilized. Based on an ion exchange mechanism, the ProteospinTM abundant serum protein depletion kit (Norgen biotek; #17300) has depleted high abundant plasma proteins. 100 mM DTT was used to achieve a 5 mM final DTT concentration, followed by gentle vortexing and incubation at 37 °C for 30 min. Next, 100 mM iodoacetamide (IAA) was used to achieve a 15 mM final IAA concentration and incubated at 25 °C (room temperature) in darkness for 30 min. 2 μL of trypsin (1 µg/µL) was added to achieve a 1:50 ratio (trypsin: protein). Samples were kept overnight at 37 °C for protein digestion. To stop the enzymatic reaction, 0.1% formic acid was added, and the pH was carefully maintained within the range of 3.0 to 4.0. The tryptic peptides were desalted using a C18 spin column (Thermo Scientific, USA). After elution, the samples were vacuum-dried and reconstituted in 0.1% (v/v) formic acid before LC–MS/MS analysis.

### Data independent acquisition for mass spectrometry (DIA)

This is a randomized and single-blinded study. Samples were run in replicates. The mass spectrometry was analyzed using an Orbitrap Fusion Lumos Tribrid Mass Spectrometer coupled with the nano-LC Easy nLC 1200 system (Thermo Fisher Scientific, Singapore). The eluted peptides were introduced into the mass spectrometer for analysis. Full scan MS1 data was acquired in the Orbitrap analyzer, operating at a resolution of 120,000 and covering a mass range of 375–2000 Da. The data acquisition utilized Xcalibur software (version 4.3.73.11, Thermo Fisher Scientific, Inc., 2019). Charge state and monoisotopic precursor ion screening were enabled to optimize the analysis. Fragmented parent ions were excluded for 40 s with an exclusion mass width of ± 10 ppm. Furthermore, a reference mass of polydimethylcyclosiloxane with m/z 445.120025 was employed for locking and enhancing the accuracy of protein mass measurement. The mass spectrometry proteomics data have been deposited to the ProteomeXchange Consortium via the PRIDE ^31^ partner repository with the dataset identifier PXD045045.

### Analysis of LCMS/MS proteomics data

Proteome Discoverer (Thermo Fisher Scientific Inc; V, 2.4.1.15) was used to process the raw files obtained from the Mass Spectrometer. To assess relative protein abundance between samples and controls, label-free quantitation (LFQ) was employed using the Proteome Discoverer processing workflow. Sequest HT tools were utilized for the search process, and MS/MS spectra annotated peak lists were compiled in the results file. Protein identification was conducted against a Uniprot (concatenated target/decoy version), Mascot database Homo sapiens (Human) sequence, and the proteome Discoverer contaminant database. Identification settings include trypsin digestion (2 missed cleavages max); minimum peptide length: 6; precursor mass tolerance: 10 ppm; fragment mass tolerance: 0.6 Da; fixed modifications: carbamidomethyl c (+ 57.021464 Da); variable modifications: oxidation of m (+ 15.994915 Da) and acetylation of protein N-term (+ 42.010565 Da). Peptides and proteins were inferred from spectrum results using the UniProt Homo sapiens (Human) database. Peptide Spectrum Matches (PSMs), peptides, and proteins were validated at a target False Discovery Rate (FDR) of 0.01 (strict) and 0.05 (relaxed). The output protein, peptide, and PSM data were exported in an Excel file, and the protein data were considered for statistical and Bioinformatics analysis.

### Pathway and network analyses of differentially expressed proteins

The dysregulated proteins were then subjected to pathway analysis using the STRING software (version 11.5, available at http://string-db.org/) and the DAVID bioinformatics tool (version 6.7, http://david.abcc.ncifcrif.gov). This analysis aimed to identify protein–protein interactions, enriched signaling pathways or networks, and categories of associated diseases. The study was performed with the parameters set to “homo sapiens” as the species and “medium confidence (0.400)” as the confidence level. The STRING analysis was conducted in “confidence” mode, utilizing the available data on protein interactions to generate meaningful insights.

### Western blot

Antibody-based validation of the C1QA (Affinity biosciences; DF7839), COMP (Affinity biosciences; DF13438), HSP90 (Affinity biosciences; AF0735), FN1 (Elabscience; E-AB-22077), and ITGA2B (Affinity biosciences; DF7456) was achieved by western blotting against their respective antibodies. β-actin (Abcam; ab8227) served as the internal control. The densitometry of the band intensities was performed through ImageJ software. The total protein was quantified using the Bicinchoninic Acid assay using BSA as a protein standard. Proteins were electrophoretically separated by 10% SDS PAGE and electrically transferred onto nitrocellulose membranes. After incubating for 2 h in blocking solution (3% BSA in TBS-T), the membranes were incubated in primary antibody β-actin, C1QA, COMP, HSP90, FN1, and ITGA2B at a concentration of 1:5000 (Antibody: 1.5% TBST) overnight at 4 °C and then into anti-rabbit HRP secondary antibody (Abclonal; ab6721) at a concentration of 1:5000 (Antibody: 1.5% TBST) for 2 h in the dark at room temperature. The nitrocellulose membrane was further stained using ECL (enhanced chemiluminescence) (Femto LUCENT, Gbiosciences, Cat: 32109), and the developed bands were quantified using the GEL-Doc apparatus (Azure Biosystem).

### ELISA

To quantitatively measure human C1QA and COMP in plasma samples obtained from individuals with Grade I and Grade II PanNET, as well as healthy controls, we utilized a commercially available human C1qA ELISA kit (GBioscience, ITLK03583) and human COMP ELISA kit (GBioscience, ITLK01678) employing a ready-to-use sandwich ELISA technique with a sensitivity of 0.059 ng/mL and 1.21 ng/mL respectively. The procedure can be summarized as follows: Monoclonal antibody-coated microwell strips were used, specifically targeting human C1qA and COMP. The first two columns were pipetted with 100 μL of standards, serially diluted. The remaining strips were prepared by adding pre-diluted plasma samples (1:1000) in a volume of 100 μL per well. The plate was sealed and incubated for 80 min at 37 °C. Following incubation, the plate was washed three times with a wash buffer and gently tapped on an absorbent pad. Biotin-conjugated anti-human C1qA and COMP antibody (100 μL) were added to each well and incubated at 37 °C for 50 min. The microplate was washed as described earlier, and then 100 μL of Streptavidin-HRP was added to all wells. The plate was sealed and incubated for 50 min at 37 °C. After six washes, 90 μL of TMB substrate solution was added to each well, and the plate was incubated at 37 °C for 30 min in darkness. The color development on the plate was monitored, and the substrate reaction was terminated by pipetting 50 μL of stop solution. An ELISA plate reader (SpectraMax i3x Multi-mode Microplate reader; Molecular Devices) measured the optical density (OD) at 450 nm. Following the manufacturer's manual, a curve-fitting process was applied to the standard curve to determine the unknown concentration of C1qA and COMP in each sample, respectively.

### Bioinformatics and statistical analysis

Proteins having at least 2 unique peptides and present in all samples were shorted for statistical and bioinformatics analysis in the free online web-based software called MetaboAnalyst (Version 5.0; (https://www.metaboanalyst.ca/MetaboAnalyst/ModuleView.xhtml)^32–34^. Samples were normalized by a median, transformed in Log (base 10), and scaled by the auto-scaling option. Fold Change (FC) for two groups comparison was calculated by dividing the normalized abundance of the case by the normalized abundance of control. The fold-change threshold of 0.67 ≥ FC ≥ 1.5 and p-value of ≤ 0.05 were considered short statistically significant biomarker candidate proteins. In MetaboAnalyst 5.0, Student’s T-test, Volcano plot, ANOVA, PLSDA, VIP score, Pearson’s correlation, Heat map, and Box-Whisker plots were analyzed.

Analysis for validation, including densitometric analysis of Western blotting and ELISA, comparing normal healthy controls with Grade I and Grade II PanNET patients, was conducted using GraphPad Prism 8.0 software. Statistical significance was determined through the unpaired student t-test, considering significance at p < 0.05. However, the data did not follow a normal distribution during statistical analysis, likely due to a small sample size and outliers. Consequently, non-parametric tests were chosen.

The ROC curve, a probability curve assessing test accuracy, played a crucial role. The AUC (Area Under the Curve) of the ROC curve reflects the measure of separability, indicating the test's capability to distinguish between classes. Plotting the ROC curve with TPR (sensitivity) against FPR (1−specificity), where TPR is on the y-axis, and FPR is on the x-axis, provides a visual representation. An outstanding test approaches an AUC of 1, signifying excellent separability, while a poor test is closer to an AUC of 0, indicating the worst separability.

### Supplementary Information

Supplementary Information.

## Data Availability

The data supporting this study’s findings are available from the corresponding authors, [SK, NR], upon reasonable request.

## Abbreviations

PanNET: Pancreatic Neuroendocrine tumors
C1QA: Complement component 1 q subcomponent, alpha polypeptide
COMP: Cartilage oligomeric matrix protein
HSP90B1: Heat-Shock Protein 90 Beta1
ITGA2B/CD41: Integrin Subunit Alpha 2b
FN1: Fibronectin 1
MSn: Multi Stage/Sequential Mass Spectrometry
PLS-DA: Partial Least Squares discriminant analysis
VIP: Variable importance in projection
