# Supplementary material for: C1QA and COMP: plasma-based biomarkers for early diagnosis of pancreatic neuroendocrine tumors
Source: Sci Rep. 2023 Nov 29;13:21021. doi: 10.1038/s41598-023-48323-x (PMC10686980; doi:10.1038/s41598-023-48323-x)
Supplement: Supplementary file 1 — Supplementary Information. [file 41598_2023_48323_MOESM1_ESM.docx]

**SUPPLEMENTARY INFORMATION FILE**

**Supplementary Table S1:** Table showing the differentially expressed proteins from Volcano Plots in Grade I PanNET patients compared to controls, and Grade II PanNET compared to Grade I PanNET patients. Bold texts represent the proteins that are common to both comparisons.

| **Gene symbols of the differentially expressed proteins obtained from volcano plots** | | | |
| --- | --- | --- | --- |
| **Grade I PanNET compared to Controls** | | **Grade II PanNET compared to Grade I PanNET** | |
| **Upregulated Proteins** | **Downregulated Proteins** | **Upregulated Proteins** | **Downregulated Proteins** |
| **MYL6** | RAN | STIP1 | CAPZA1 |
| **FLNA** | IGHA2 | IGHV3-23 | **MYL6** |
| **MYL9** | ANGPTL6 | RGS10 | CLTC |
| LIMS1 | V387 | SEP02 | **FLNA** |
| FCN3 | IGF1 | NAP1L1 | SKAP2 |
| **ACTN1** | V472 | HSPB1 | VCP |
| **TUBB** | MGAT1 |  | ATP5A1 |
| **FN1** | **COMP** |  | HSP90B1 |
| **MYH9** | **FKBP3** |  | YWHAE |
| **ITGA2B** | MPP1 |  | COX6B1 |
| LPA | SAR1A |  | FYB |
| HIST1H2BN; | V622 |  | ATP5B |
| VASP | UNC13D |  | SLC25A6;SLC25A5;SLC25A4 |
| MYL6 | IGHV3-23 |  | **ITGA2B** |
| FLNA | HNRNPA1;HNRNPA1L2 |  | CFHR3 |
| MYL9 | FBLN1.1 |  | MNDA |
| LIMS1 | DIAPH1 |  | **ACTN1** |
| FCN3 | HNRNPC |  | **MYH9** |
| ACTN1 | GPX3 |  | TUBA4A |
| TUBB | GNPTG |  | **TUBB** |
| FN1 | V469 |  | PFKP |
| MYH9 | CPNE1 |  | ITGB3 |
|  | ADAMTSL4 |  | C1QB |
|  | RDX |  | SERPINA3 |
|  | OAF |  | KPNB1 |
|  | IGLC6 |  | **C1QA** |
|  | **C1QA** |  | BTD |
|  |  |  | TMOD3 |
|  |  |  | PDIA5 |
|  |  |  | GC |
|  |  |  | EEF2 |
|  |  |  | VIM |
|  |  |  | IDH2 |
|  |  |  | HNRNPU |
|  |  |  | TGFB1I1 |
|  |  |  | HIST1H4A |
|  |  |  | **MYL9** |
|  |  |  | CAPZB |
|  |  |  | GNAI2 |
|  |  |  | STXBP2 |
|  |  |  | HCLS1 |
|  |  |  | EHD1 |
|  |  |  | TUBA1B |
|  |  |  | APOB |
|  |  |  | ASPN |
|  |  |  | ITIH2 |
|  |  |  | MYL12A;MYL12B |
|  |  |  | PCYOX1 |
|  |  |  | ACTG1 |
|  |  |  | HSPA5 |
|  |  |  | TPM3 |
|  |  |  | FCN2 |
|  |  |  | APOA2 |
|  |  |  | NCK2 |
|  |  |  | ORM1 |
|  |  |  | F12 |
|  |  |  | PDIA6 |
|  |  |  | HNRNPK |
|  |  |  | YARS |
|  |  |  | **FKBP1A;FKBP12-Exin;FKBP12-Exip2** |
|  |  |  | SCGB3A1 |
|  |  |  | **COMP** |
|  |  |  | HGFAC |
|  |  |  | FGA |
|  |  |  | **FN1** |
|  |  |  | PDCD10 |
|  |  |  | PCOLCE |
|  |  |  | TLN1 |
|  |  |  | SPP2 |


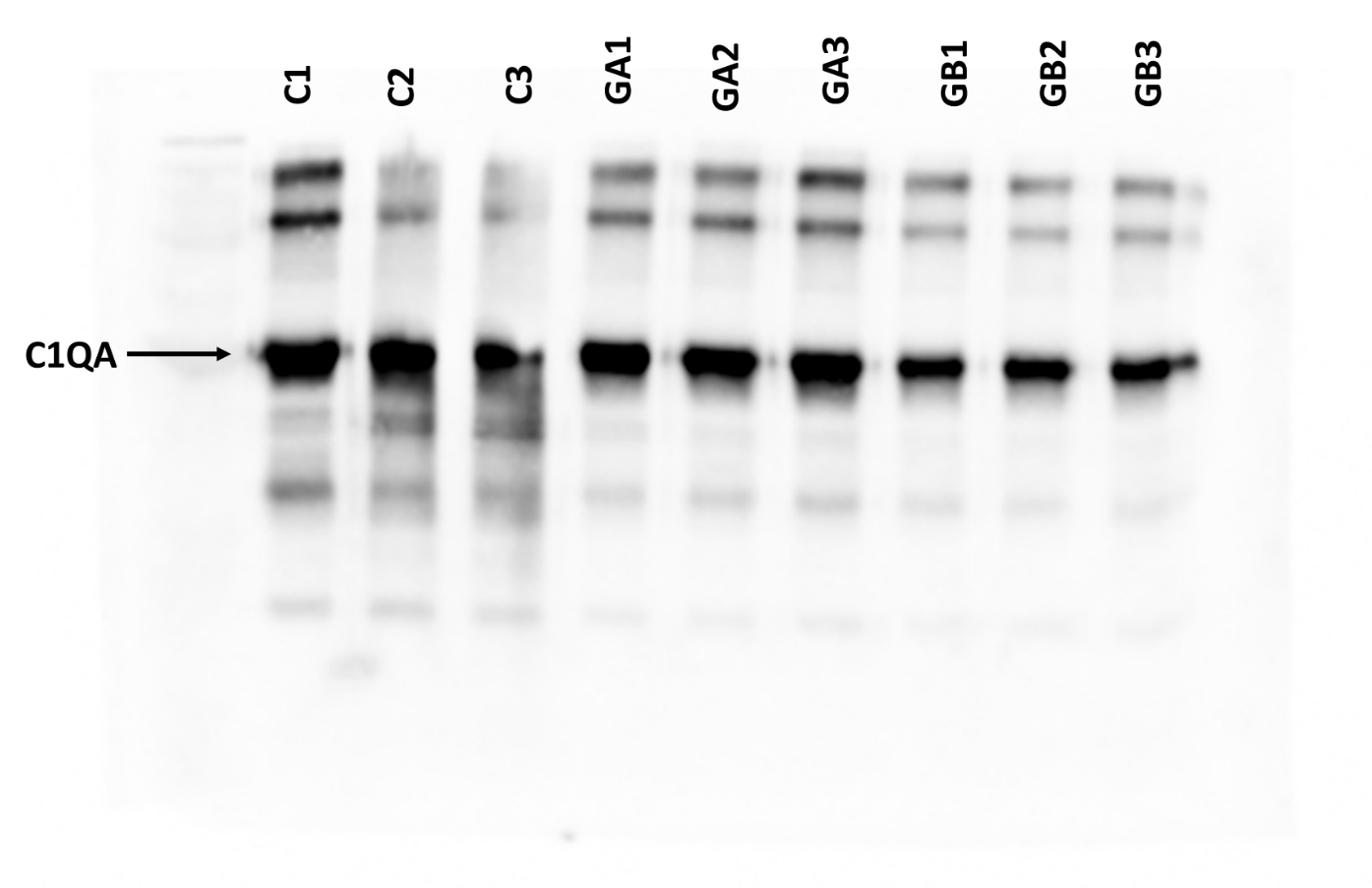


**Figure S2:** **Western blot of anti-C1QA** **in Healthy Controls (C1, C2 and C3), Grade I PanNET (GA1, GA2 and GA3), Grade II PanNET (GB1, GB2 and GB3).**

**
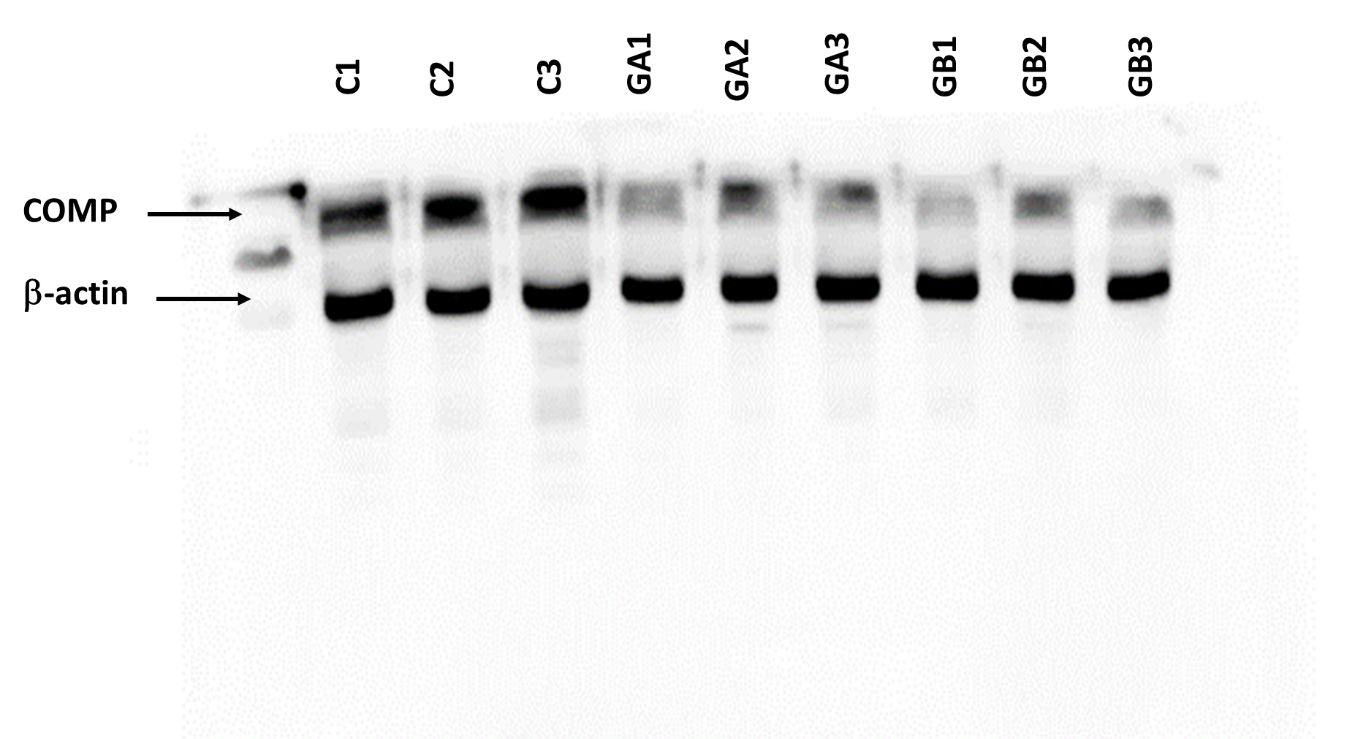
**

**A**

**
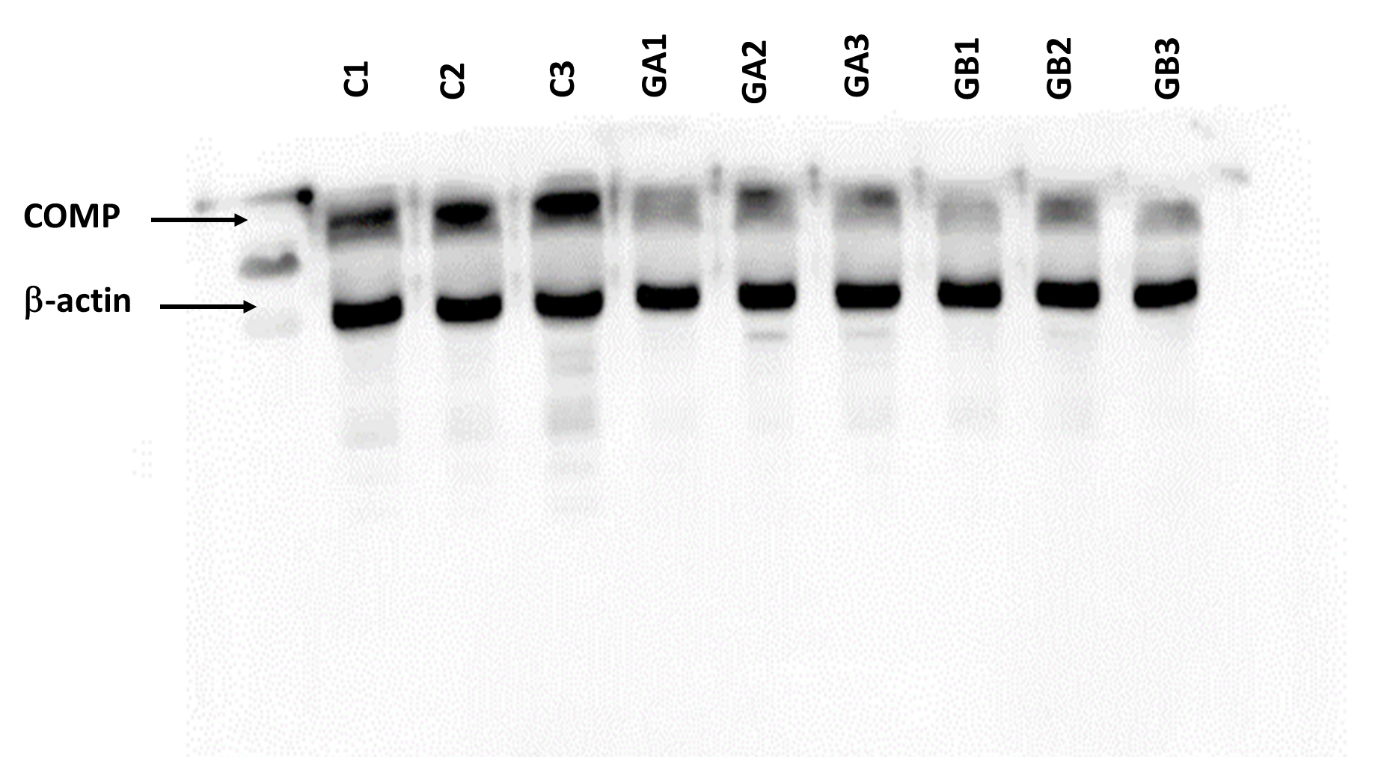
**

**B**

**Figure S3: Western blot of anti-COMP and** **anti-β-actin in Healthy Controls (C1, C2 and C3), Grade I PanNET (GA1, GA2 and GA3), Grade II PanNET (GB1, GB2 and GB3), with different exposure settings (A & B).**

**
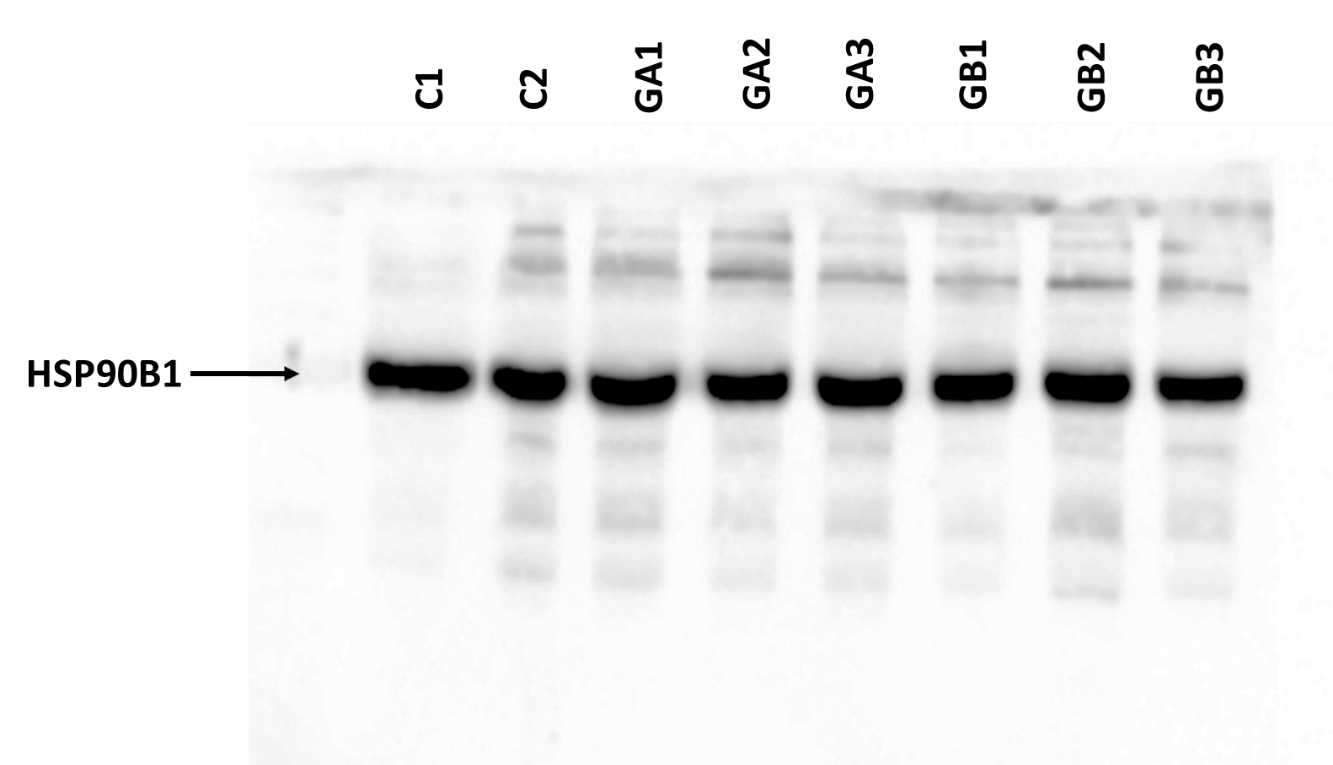
**

**Figure S4: Western Blot of anti-HSP90B1 in Healthy Controls (C1 and C2), Grade I PanNET (GA1, GA2 and GA3), Grade II PanNET (GB1, GB2 and GB3).**

**
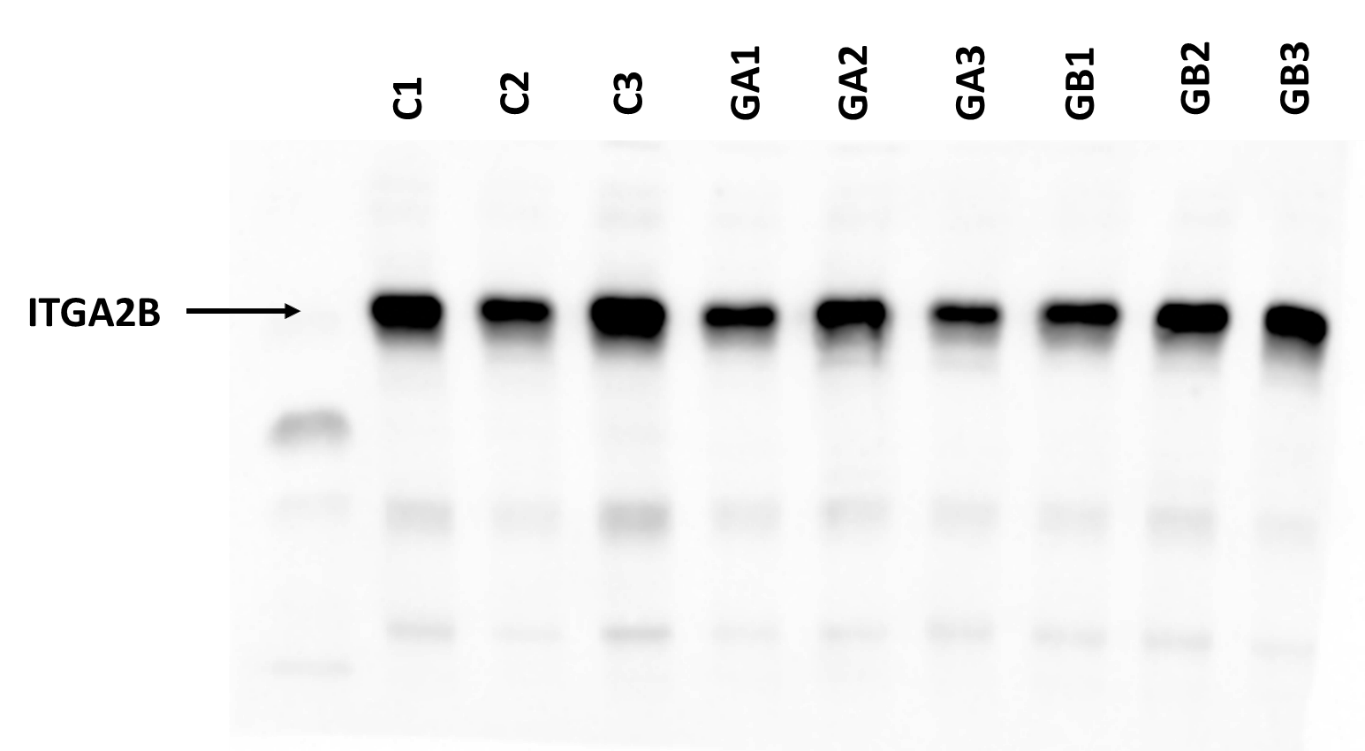
**

**Figure S5: Western blot of anti-ITGA2B in Healthy Controls (C1, C2 and C3), Grade I PanNET (GA1, GA2 and GA3), Grade II PanNET (GB1, GB2 and GB3).**

**
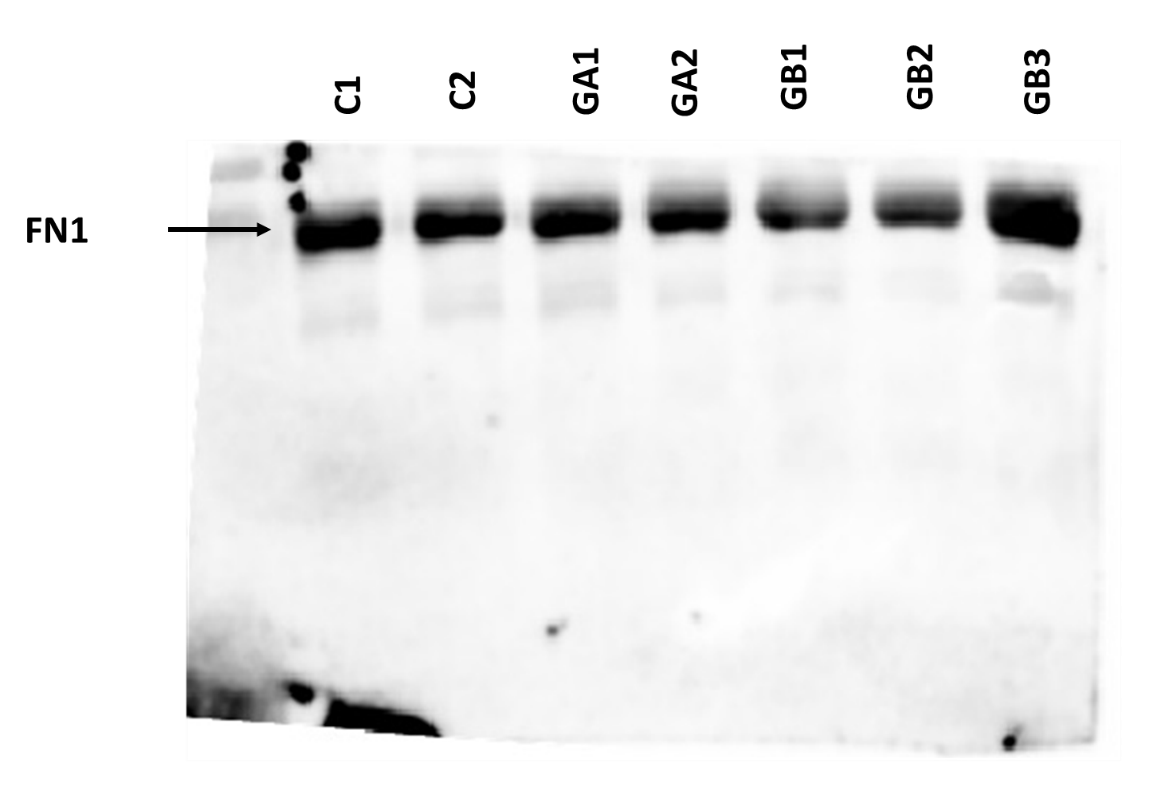
**

**Figure S6: Western Blot of anti-FN1 in Healthy Controls (C1 and C2), Grade I PanNET (GA1, GA2 and GA3), Grade II PanNET (GB1, GB2 and GB3).**

**
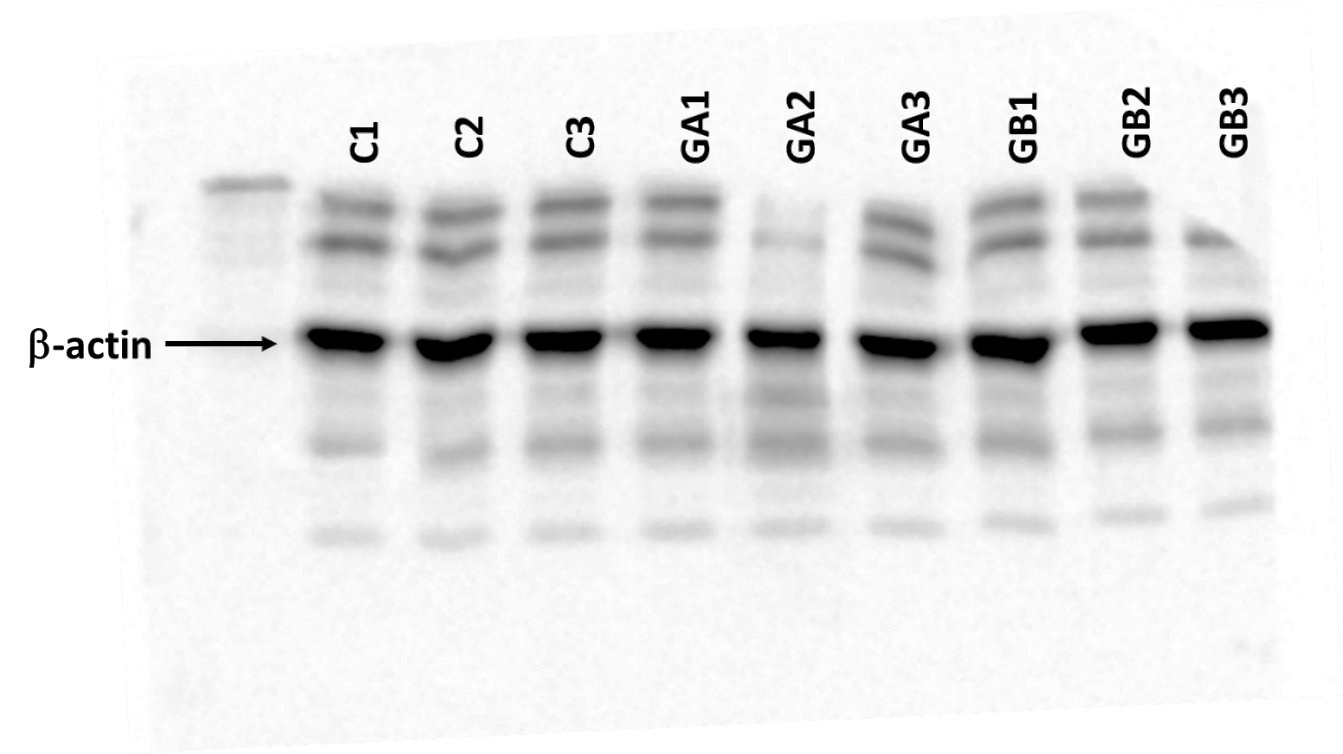
**

**Figure S7: Western Blot of anti-Beta Actin in Healthy Controls (C1, C2 and C3), Grade I PanNET (GA1, GA2 and GA3), Grade II PanNET (GB1, GB2 and GB3).**
